# Wastewater-Based Epidemiology as a Tool to Detect SARS-CoV-2 Circulation at the Community Level: Findings from a One-Year Wastewater Investigation Conducted in Sicily, Italy

**DOI:** 10.3390/pathogens12060748

**Published:** 2023-05-23

**Authors:** Carmelo Massimo Maida, Fabio Tramuto, Giovanni Maurizio Giammanco, Roberta Palermo, Walter Priano, Simona De Grazia, Giuseppa Purpari, Giuseppina La Rosa, Elisabetta Suffredini, Luca Lucentini, Mario Palermo, Walter Pollina Addario, Giorgio Graziano, Palmira Immordino, Francesco Vitale, SARI Collaboration Group, Walter Mazzucco

**Affiliations:** 1 Department of Health Promotion, Mother and Child Care, Internal Medicine and Medical Specialties “G. D’Alessandro”, University of Palermo, Via del Vespro 133, 90127 Palermo, Italy; 2 Clinical Epidemiology Unit, Regional Reference Laboratory of Western Sicily for the Emergence of COVID-19, University Hospital “P. Giaccone”, Via del Vespro 133, 90127 Palermo, Italy; 3 Istituto Zooprofilattico Sperimentale della Sicilia “A. Mirri”, Via Marinuzzi, 90129 Palermo, Italy; giuseppa.purpari@izssicilia.it; 4 Istituto Superiore di Sanità (ISS), Viale Regina Elena 299, 00161 Rome, Italy; giuseppina.larosa@iss.it (G.L.R.);; 5 Regional Health Authority of Sicily, Via Vaccaro 5, 90145 Palermo, Italy

**Keywords:** wastewater, SARS-CoV-2, surveillance, wastewater-based epidemiology, active cases, COVID-19

## Abstract

Wastewater-based epidemiology is a well-established tool for detecting and monitoring the spread of enteric pathogens and the use of illegal drugs in communities in real time. Since only a few studies in Italy have investigated the correlation between SARS-CoV-2 in wastewater and the prevalence of COVID-19 cases from clinical testing, we conducted a one-year wastewater surveillance study in Sicily to correlate the load of SARS-CoV-2 RNA in wastewater and the reported cumulative prevalence of COVID-19 in 14 cities from October 2021 to September 2022. Furthermore, we investigated the role of SARS-CoV-2 variants and subvariants in the increase in the number of SARS-CoV-2 infections. Our findings showed a significant correlation between SARS-CoV-2 RNA load in wastewater and the number of active cases reported by syndromic surveillance in the population. Moreover, the correlation between SARS-CoV-2 in wastewater and the active cases remained high when a lag of 7 or 14 days was considered. Finally, we attributed the epidemic waves observed to the rapid emergence of the Omicron variant and the BA.4 and BA.5 subvariants. We confirmed the effectiveness of wastewater monitoring as a powerful epidemiological proxy for viral variant spread and an efficient complementary method for surveillance.

## 1. Introduction

Over the past 30 years, many studies have shown that wastewater testing can provide an affordable estimate of the burden of infectious diseases at the population level [1,2,3,4,5]. Since the beginning of the COVID-19 pandemic, numerous pilot studies have shown the use of wastewater-based epidemiology (WBE) as a tool to monitor the circulation of SARS-CoV-2, highlighting the strong link between environmental and clinical frameworks [6,7,8,9,10,11,12,13,14]. In November 2020, the World Health Organization Regional Office for Europe [15] organized an expert consultation on the surveillance of SARS-CoV-2, which led the European Commission to adopt a common approach to establish systematic surveillance of SARS-CoV-2 variants in wastewater in the European Union [16]. Nowadays, wastewater monitoring of SARS-CoV-2 represents an important complementary tool to the existing public health surveillance systems worldwide based on newly reported clinical cases of COVID-19 and provides information on infection trends in a specific community, irrespective of the availability and access to resources for individual testing. Moreover, recent research has shown that trends in wastewater SARS-CoV-2 concentrations are highly predictive of the number of COVID-19 cases, allowing the earlier detection of SARS-CoV-2 in the community compared to clinical-based surveillance systems [17,18,19,20]. Thus, wastewater monitoring may serve as an early warning system since an infected person may shed SARS-CoV-2 RNA in stool and respiratory droplets about 3–5 days before the onset of symptoms [21]. Surveillance based on clinical testing typically captures individuals specifically tested for SARS-CoV-2; therefore, it depends on testing capacity, reagent costs, properly equipped laboratory facilities, expert hands, and the population’s compliance to be tested [22]. Thus, environmental surveillance could complement the potential under-reporting of clinical surveillance by supporting the monitoring of trends in COVID-19 incidence and transmission in a community [23]. From a public health perspective, an alert of increasing cases obtained from wastewater surveillance can provide health departments with critical time to decide on the best allocation of resources and preventive measures [24]. Furthermore, sewage surveillance provides cost-effective and efficient monitoring of the entire population’s health within a watershed, even when a robust syndromic surveillance system is lacking in the community [25]. In Italy, integrated microbiological and epidemiological surveillance for COVID-19 continuously and systematically collects, compares, and analyzes information on all clinically suspected SARS-CoV-2 infections confirmed by regional reference laboratories [26]. Clinical surveillance represents a necessary and valuable observation tool for estimating the impact and evolution of the epidemic, and it offers valid support in decision making for public health preparedness and response. The information in the COVID-19 national surveillance system comes from a complex data stream that starts at the local level retrieving results of antigenic or molecular diagnostic testing of suspected cases in those under home isolation or those that are hospitalized. Results of confirmed cases are communicated to the local health authorities, who coordinate the collection of detailed data on each positive individual from hospitals, general practitioners, and general pediatricians. COVID-19 data are then shared with the national epidemiological surveillance system coordinated by the National Institute of Health (NIH). In July 2020, the NIH launched a nationwide wastewater pilot monitoring program (SARI project) to investigate the spread of SARS-CoV-2 in tourist locations during the summer, autumn, and winter [27]. This study aimed to estimate the potential of an environmental surveillance system in detecting SARS-CoV-2 variants/subvariants spread and to compare these data with those retrieved by the clinical, epidemiological surveillance system already in place. Therefore, we conducted a correlation analysis comparing the concentration of SARS-CoV-2 RNA detected during a 12-month period in the wastewater of 13 different municipalities located in Sicily to the cumulative prevalence of COVID-19 provided by the officially integrated surveillance system operating in Italy, based on the results of clinical testing performed in the same period. Moreover, a pre–post correlation analysis was performed to assess the reliability of the epidemiological and virological surveillance system after ceasing the COVID-19 pandemic emergency state declared on 1 April 2022 [28], dividing the study period into two semesters.

## 2. Materials and Methods

### 2.1. Study Design and Sample Collections

**Figure 1:**
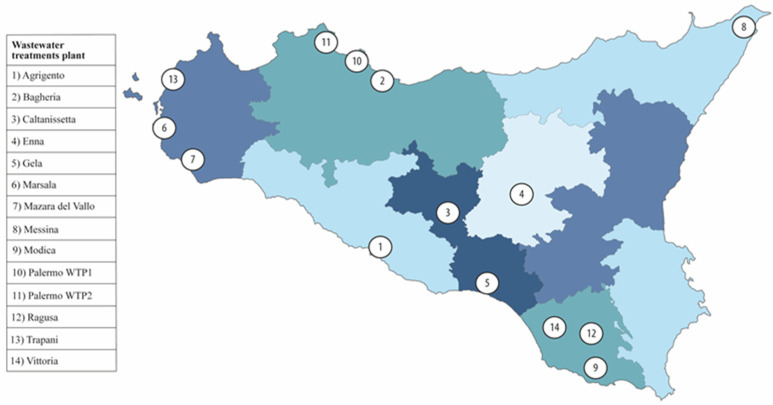
Map showing the locations of the wastewater treatment plants included in the COVID-19 environmental surveillance system.

The present observational study was conducted in Sicily (Italy), the largest and most populous island in the Mediterranean Sea, accounting for about 5 million resident inhabitants [29]. Fourteen wastewater treatment plants (WWTPs) located in thirteen different Sicilian municipalities and serving a cumulative population of 1,187,059 inhabitants (each serving a range of 12,000 to 314,973 inhabitants; 23.7% of the total population of Sicily) were included in the study. Raw 24-h composite wastewater samples (n = 632) were collected weekly for 12 months between 1 October 2021, and 30 September 2022 (39th week of the year) using automatic sampling devices. Further information about the location and the characteristics of WWTPs is provided in Figure 1 and Table 1. 

The collected samples were refrigerated and transferred to three different laboratories, identified by the Regional Health Authority of Sicily, stored at +4 °C, and then tested for SARS-CoV-2 RNA within 24 h from sampling. All laboratories participating in the study received reference materials to perform Real-time PCR and underwent proficiency testing organized by the NIH to evaluate their performance in executing the specific assay.

### 2.2. Laboratory Methods

Laboratory analyses were performed according to the national protocol designed for the SARI network [30]. The materials and methods section below provides a thorough explanation of all the analytical phases, despite the protocol being published in Italian.

#### 2.2.1. Virus Concentration

All samples underwent a 30 min treatment at 56 °C to ensure the safety of laboratory personnel and the environment [6]. Then, each sample was concentrated using a polyethylene glycol (PEG)-based procedure, according to the protocol by Wu et al. [31] with minor modification. Briefly, wastewater samples (45 mL) were centrifuged at 4500× *g* for 30 min; after centrifugation, 40 mL of sample was mixed with polyethylene glycol 8.000 8% (wt/vol) and NaCl (0.3 M) (both supplied by Sigma-Aldrich, St. Louis, MO, USA) and spiked with a known amount of Murine Norovirus or Mengovirus, used as a process control. After a centrifugation step at 12,000× *g* for 2 h, the viral pellet was resuspended in 2 mL of NucliSENS Lysis Buffer reagent (bioMerieux, Marcy-l’Étoile, France) for subsequent RNA extraction.

#### 2.2.2. RNA Extraction

RNA extraction was performed using a semi-automated method with magnetic silica beads (supplied by bioMerieux, Marcy l’Etoile, France). After an incubation step at room temperature for 20 min, 100 μL of magnetic silica beads were added, and, after a further 10 min incubation, an automated procedure was performed using a nucleic acid purification system (Auto-Pure96, All Sheng Instruments, Zhejiang, China) or a Nuclisens MiniMag system (bioMerieux, Marcy l’Etoile, France). The extracted nucleic acids were then purified from potential PCR inhibitors using the OneStep PCR Inhibitor Removal Kit (Zymo Research, CA, USA).

#### 2.2.3. RT-qPCR

All RT-qPCR assays for SARS-CoV-2 targeting the ORF1b (nsp14) were conducted on the QuantStudio 6 and 7 Flex Real-Time PCR System (ThermoFisher Scientific, Waltham, MA, USA). In detail, the thermal protocol was carried out as follows: reverse transcription at 50 °C for 30 min, denaturation at 95 °C for 10 min, followed by 45 cycles of 95 °C for 15 s and 60 °C for 45 s. The reaction mixture (final volume: 15 µL) consisted of 3.9 µL of Master Mix (QuantiNova Pathogen kit - Qiagen, CA, USA), 0.45 µL of each primer (30 µM), 0.3 µL of probe (10 µM), water to the volume of 10 µL, and 5 µL of RNA template. All reactions were run in quadruplicate. Molecular biology water served as a non-template control. Reactions were considered positive only if the threshold for positivity was passed within 40 cycles (Ct < 40). To determine the amount of dsDNA SARS-CoV-2 present, 10-fold dilutions were used, ranging from 1.0 to 1.0 × 10^5^ genomic copies (GC) per reaction, provided by the NIH. Linear regression of cycle threshold (Ct) values versus the Log10 concentration of the standard was used to generate RT-qPCR standard curves, which were then used to convert Ct values into ORF1b copies/μL per reaction.. SARS-CoV-2 GC/L in wastewater was obtained according to the following formula: C (RNA GC/ μL) × 100 (total volume of RNA of the extracted sample) × 25 (ratio factor between analyzed volume and reference volume of 1 L). The results were also evaluated in GC/day/inhabitant according to the following formula: flow rate of WWTP in 24h (m^3^) × GC (m^3^)/equivalent number of inhabitants served by the WWTP. Verification of PCR inhibition was performed as a quality parameter of the determinations. To verify the inhibition, the PCR Ct value obtained from the sample added with 1.0 × 10^3^ GC/μL RNA SARS-CoV-2 nsp14 provided by the NIH was compared with the PCR Ct value of water for a molecular biology sample added with 1 μL of the same RNA according to the following formula: ΔCt = Ct (sample + control RNA) − Ct (water + control RNA). The sample was considered acceptable if ΔCt was ≤2. Before performing sample analysis, the LoD was determined by spiking wastewater extracts with dilutions of dsDNA SARS-CoV-2 nsp14 solutions (provided by the NIH) at concentrations of approximately 1000, 100, 50, 20, 10, 2, and 1.0 GC/μL. Ten replicates of each dilution were tested. The LoD was the lowest concentration at which all ten replicates were positive. The assay had a LoD of 2 GC/μL. To assess the concentration/extraction efficiency of the method, 100 μL of a process control virus solution (Murine Norovirus at 2.5 × 10^3^ GC/μL) was added to 40 mL of each wastewater sample prior to concentration. Samples were then concentrated and extracted. For the PCR assays, serial 10-fold dilutions of a stock solution of the process control virus were used to generate standard curves. The Ct values of the reactions of samples spiked with the process control virus were compared with the Ct value of the reaction containing the undiluted process control virus, and the concentration/extraction efficiency (%) was calculated according to the following formula: 10 (ΔCt/m) × F × 100 (ΔCt = Ct sample − Ct undiluted solution of control process virus; m—slope of the control process virus standard curve; F—fraction of the initial sample processed). The sample was considered acceptable if the concentration/extraction efficiency was ≥1% [30].

#### 2.2.4. Flash Surveying the SARS-CoV-2 Variants

A study on SARS-CoV-2 circulating variants was conducted monthly on a national basis. At a regional level, wastewater samples were collected in all Sicilian WWTPs and preliminarily processed by the regional laboratories belonging to the SARI network. Purified RNAs were shipped frozen on dry ice to the NIH for subsequent sequencing analysis [32].

#### 2.2.5. Clinical Data Sources

Sicilian COVID-19 cases, and their relevant clinical data, were recorded in the web-based integrated national surveillance platform established by the NIH [26]. SARS-CoV-2-positive patients were considered eligible if they met the following inclusion criteria: resident in Sicily (or temporarily domiciled in Sicily) and having a laboratory-confirmed SARS-CoV-2-positive result from nasal, pharyngeal, or nasopharyngeal swabs between 1 October 2021 and 31 August 2022.

#### 2.2.6. Statistical Analyses

Data obtained from wastewater sample analyses and data retrieved from the integrated national surveillance platform—providing daily SARS-CoV-2 epidemiological data for each municipality, including prevalence of infection, number of hospitalizations, deaths, and ICU admissions—were collected through Microsoft Excel (version 2010). The total number of active infections in all municipalities served by each WWTP was calculated by considering all individuals diagnosed with a SARS-CoV-2 infection through a positive swab within 15 days of the wastewater sampling date and not yet declared cured after a negative swab as “active”. The two databases were merged and analyzed with RStudio (version 4.2.2). New time-dependent variables were created using the sampling date and WWTP location as key variables; prevalence was set at time 0, 7, and 14 (t0, t7, and t14, respectively) days after the sampling date. The Shapiro–Wilk test was carried out to check for the normality of each continuous variable. Furthermore, when more than one sample was taken within the same week of the year and across weeks—even if samples were collected from different municipalities—data on the amount of GC/L and the prevalence of SARS-CoV-2 cases at t0, t7, and t14 were aggregated into means. Pearson’s correlation test, log-linear regression analyses, and significance tests were carried out to compare the mean weekly prevalence of SARS-CoV-2-positive cases with the mean weekly amount of genomic copies/L derived from wastewater analysis at different time periods (0 to 6 months, 7 to 12 months, and 12 comprehensive months) and different times; t0 (intended as the week the sample was taken), t7, and t14 ahead of t0, respectively. Spearman’s correlation test was performed to compare discrete variables. A correlation analysis was performed between the weekly amount of SARS-CoV-2 GC/L and the other variables enlisted, such as the number of hospitalizations, deaths, and ICU admissions, cumulatively considered as the “severe clinical outcome” variable. The significance level chosen was a *p*-value < 0.05 (two-tailed).

## 3. Results

**Figure 2:**
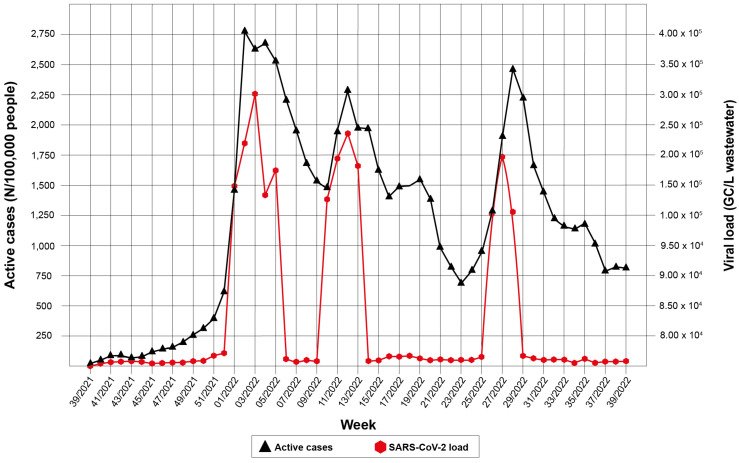
Weekly trends in viral load detected from the 14 WWTPs and prevalence of active cases in the Sicilian municipalities during the 12-month surveillance.

Overall, 95.7% (n = 605/632) of the wastewater samples were positive for SARS-CoV-2 RNA over the 12-month study period. The recovery rate of SARS-CoV-2 from wastewater may have varied depending on the physic-chemical properties of the wastewater samples (mean = 23.8%; range = 1.0–100.0%; 95%C.I. = 2.4). The PCR inhibition rate of the sewage samples was 0.6 Ct (range 0.0–8.3 Ct; 95%C.I. = 0.01 Ct) compared to a SARS-CoV-2 RNA control of known concentration in PCR water grade. Based on these results, SARS-CoV-2 RNA loads in wastewater were compared to the number of COVID-19 infections recorded in the corresponding area. In the entire study period, the total amount of SARS-CoV-2 shed weekly by the infected subjects, regardless of their clinical status (symptomatic, paucisymptomatic, and asymptomatic) ranged from 0.0 to 5.9 × 10^7^ GC/day/inhabitant, corresponding to 0.0 and 2.8 × 10^5^ GC/L in wastewater. Two epidemic waves of infections have been observed in the population, the first between late December and the end of March (week 52/2021–13/2022), with an average wastewater viral load of 3.2 × 10^5^ GC/L (range 1.0 × 10^5^–1.2 × 10^6^), and the second between the end of June and the end of July 2022 (weeks 26/2022–30/2022), with an average viral load of 1.1 × 10^5^ (range 4.4 × 10^4^–1.6 × 10^5^). Data on viral load and active cases in each municipality during the one-year survey are relatively homogeneous, which suggests a similar circulation of the virus in all the municipalities covered by the study. Figure 2 reports the relationship between the total number of active cases in the population (primary y-axis) and the SARS-CoV-2 load in sewage (secondary *y*-axis) per week of the year of the observation time (*x*-axis).

**Figure 3:**
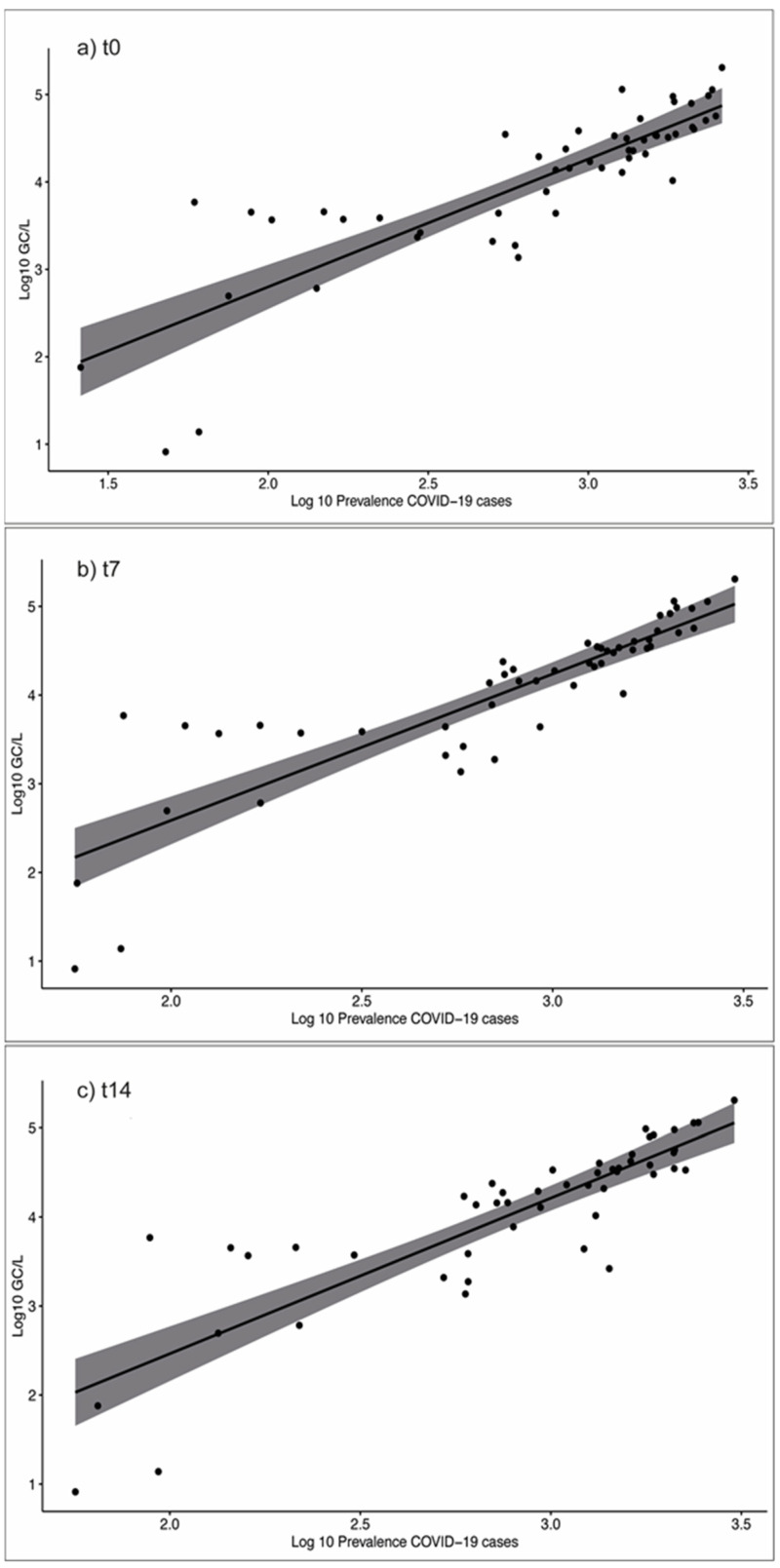
Correlation between the viral load detected in wastewater and the prevalence of active cases reported in the communities during the 12-month COVID-19 surveillance at t0 (**a**) (R = 0.85; *p*-value < 0.001), t7 (**b**) (R = 0.87; *p*-value < 0.001), and t14 (**c**) (R = 0.86; *p*-value < 0.001). Log10 GC/L: viral load in wastewater; Log10 Prevalence COVID-19 cases: prevalence of active cases.

The cumulative COVID-19 epidemic curve observed was well overlapped with the SARS-CoV-2 RNA load from wastewater samples, with an increasing trend of SARS-CoV-2 in wastewater samples comparable to the rise of active cases in the population. Every first week of each month of the survey, SARS-CoV-2 variants/subvariants were characterized. In particular, the first peak of active cases, recorded in the weeks 52/2021–13/2022 was preceded by and associated with the rapid spread of the Omicron variant, observed in 5/14 WWTPs between the weeks 45/2021 and 51/2021 [33] and in 8/13 cities by week 7/2022 [34]. The second peak of infection, recorded in the weeks 26/2022–30/2022, overlapped with the spread of subvariants BA.4 and BA.5, found in 7/13 municipalities in the same time [35]. To assess whether the number of active cases of COVID-19 in the population correlated with the number of genomic copies of SARS-CoV-2 in wastewater, weekly correlation analyses were performed for all municipalities included in the study. As shown in Table 2, analysis of the 12 months of the study revealed a significant correlation between the viral load in wastewater and the number of active cases (R = 0.85; *p*-value < 0.001) (Figure 3) and between the viral load in wastewater and the severe clinical outcomes (R = 0.90; *p*-value < 0.001).

To verify if there were differences between the wastewater sampling date and the date on which the prevalence of active cases was determined, the correlation between active cases and the viral load in the wastewater was evaluated by applying time lags of 7 and 14 days following the date of wastewater sampling. The correlation during the period 0–12 months was highly positive for all lags considered (R = 0.85 for t0; R = 0.87 for t7; R = 0.86 for t14; *p*-value < 0.001). Significant differences emerged, however, when considering the two semesters of the study period (i.e., months 0–6 and 7–12). In the first semester, the correlation between the viral load in the wastewater and the active cases was higher (R = 0.90; *p*-value < 0.001) than in the second semester (R = 0.77; *p*-value < 0.001), and the same trend was also observed in relation to the severe clinical outcomes (R = 0.79; *p*-value < 0.001 and R = 0.75; *p*-value < 0.001, respectively). To clarify if the trend shown for the lag of 7 and 14 days in the two separate semesters was the same as that recorded in the period of 0–12 months, a further correlation analysis was carried out between active cases and GC/L in wastewater. The best correlation was observed at t0 for the first semester and at t7 for the second semester, even if the correlation remained highly positive 7 days after sampling, demonstrating that the viral load in wastewater could anticipate active cases in the population by at least one week.

## 4. Discussion

The detection of SARS-CoV-2 RNA in the WWTPs of 13 Sicilian municipalities with different population sizes (approximately ranging from 27,586 to 670,000 inhabitants) was used to assess whether WBE could represent a good proxy for the early spread of the virus into populations. Although the viral RNA recovery efficiency does not seem high (95% C.I. = 2.4%), it is important to recognize that several studies have investigated different concentration/extraction methods and different viral surrogates, making it difficult to make direct comparisons and generalizations [36]. Each viral surrogate shows different interactions with wastewater depending on the characteristics of the wastewater and those of the viral surrogates, which may exhibit different partitioning/degradation characteristics. Recent studies investigating surrogate virus recovery following similar PEG concentrations reported variable results from <6% for murine hepatitis virus (MHV) [37] to 57% for Escherichia virus bacteriophage MS2 [38].

Our findings showed that SARS-CoV-2 RNA was detected in all the monitored sites in small and large treatment plants. Two epidemic waves of SARS-CoV-2 occurred in Sicily between 1 October 2021 (week 39/2021) and September 30, 2022 (week 39/2022). The first wave showed a high intensity of viral circulation, peaking in the week 02/2022, with an average cumulative number of weekly active cases of 2766.97 per 100,000 inhabitants (range 2017.41–3515.53/100,000). The determination of the viral load in wastewater showed an average minimum value of 1.3 × 10^5^ GC/L for the same week in all the municipalities under investigation, while when evaluating the average concentration, the viral load peak in Sicily was reached in the third week of 2022, with a value of 3.0 × 10^5^ (range 4.1 × 10^3^–7.6 × 10^5^), therefore demonstrating that the maximum number of infections recorded during the first wave overlapped with the detection of viral RNA in wastewater, as indicated by the correlation analysis, which showed high values (R = 0.90; *p*-value *<* 0.001), especially in the first semester of the study period.

The second wave of infection showed a significant increase in active cases, peaking in week 28/2022, with an average number of active cases of 2407.08 per 100,000 inhabitants (range 1314.87–3468.48/100,000). In this case, an average value of 5.6 × 10^3^ GC/L occurred in all the municipalities included in the study in week 26/2022. Accordingly, evaluating the average concentration, the peak of the viral load was reached in the week preceding the peak of infections (27/2022) with a value of 1.0 × 10^5^ (range 3.4 × 10^3^–3.1 × 10^5^), thus showing that wastewater analysis can predict the peaks of infections at the population level.

Among the different variants of SARS-CoV-2 circulating over the study period, WBE showed a rapid and complete replacement of the Delta variant (detected until week 48/2021) by the Omicron variant, as highlighted in the “Ad hoc survey on B.1.1.159 on SARS-CoV-2 in urban wastewater in Italy” [33] conducted by the NIH from week 49/2021 to week 51/2021 in several Sicilian municipalities. This rapid spread of the B.1.1.159 variant was correlated with the maximum number of active cases observed in the weeks between 1/2022 and 5/2022 and was followed by a significant increase in viral load in wastewater in all Sicilian municipalities. The rapid spread of the Omicron variant and subvariants was also detected, confirming the effectiveness of wastewater monitoring as a powerful epidemiological proxy for viral circulation and variants spread. Therefore, WBE can be proposed as an efficient alternative/complementary method for surveillance purposes.

The correlation between the viral load in the wastewater and the active cases proved to be very high throughout the study period. These findings align with other studies investigating the relationship between viral load in wastewater and active cases of COVID-19 [39,40,41]. When considering the two semesters under study, we recorded a decrease in the correlation values in the second semester. The second semester of surveillance ranged from April to September, a period of the year when the climatic conditions in Sicily are considerably warmer compared to the winter periods. Increased temperatures could lead to a lower environmental persistence of SARS-CoV-2 RNA, probably due to greater degradation of viral nucleic acid in wastewater, with the consequent reduction in viral load, as previously documented by some authors [42]. The decrease in correlation was accompanied by lower levels of the viral load per active cases, despite their number increasing in the same summer period. Generally, viral load in wastewater is affected by factors that can be controlled only partially, such as the sampling/transport of samples, the variable chemical-physical characteristics of the wastewater, and the possible under-reporting of active cases of COVID-19.

## 5. Conclusions

Monitoring SARS-CoV-2 in wastewater can serve as an effective surveillance tool to describe the evolution of the COVID-19 pandemic at the community level. It can represent complementary epidemiological indicators since it can offer, with a single measurement, the ability to reflect the state of the pandemic in the general population and to predict the spread of the infection. This indicator can integrate data from the ongoing virological surveillance of COVID-19, providing reliable estimates of the spread of SARS-CoV-2, including both symptomatic and asymptomatic individuals, burdening the population-level prevalence of COVID-19 and the real dimension and trends of the pandemic in a population. Therefore, WBE can be helpful for monitoring the effectiveness of public health interventions for the control of the pandemic. The surveillance of wastewater samples may also serve as a valuable early warning in introducing novel SARS-CoV-2 variants and for the eventual re-emergence of their spread in the post-pandemic period. However, the usefulness of WBE and environmental surveillance has encountered several obstacles, such as the need for standardized methods, prompt information and communication between surveillance operators and public health decision-makers, as well as the correct interpretation of the results generated by such a community surveillance approach [18]. Moreover, the detection and quantification of SARS-CoV-2 in the environment may be limited by false negative results or the underestimation of the concentration due to the complexity of the environmental matrix, structural differences of the sewage networks and plants, or delays in data collecting times, thus deserving further exploration.

COVID-19 environmental surveillance is evolving rapidly, and recent interim guidance has indicated SARS-CoV-2 environmental surveillance as a complement to clinical surveillance [43,44], offering and adding value to healthcare sector decision making in the COVID-19 response [45]. WBE can detect hotspots, provide early warnings, and determine the prevalence of various diseases. On the other hand, clinical diagnostic tests can be used to diagnose positive patients and implement mass vaccination and quarantine measures to limit direct, indirect, or close contact. However, clinical diagnostic tests need to improve their analyses’ speed, sensitivity, and portability to be cost-effective and efficient. It is also noteworthy that the presence of SARS-CoV-2 in a community can be detected earlier by WBE than by clinical diagnostic tests. Hence, dual monitoring of COVID-19, using WBE and clinical diagnostic tests, will help control the spread and threat of the COVID-19 pandemic [22]. We assume that clinical-based surveillance systems may have under-reported cases due to several factors: an increase in the number of individuals with unnotified SARS-CoV-2 infection, probably due to clinical self-testing; the reduced severity and duration of symptoms despite sustained virus shedding in the post-symptomatic phase; and the failure to test and detect asymptomatic individuals [19,46,47,48]. However, it should also be noted that an absolute comparison between the prevalence of SARS-CoV-2 infection in the population and the concentration of SARS-CoV-2 RNA in wastewater may raise some issues, as the reported prevalence is highly dependent on the testing methods and capacity [49]. Moreover, WBE could acquire increased relevance to provide a prompt picture of virus circulation in the post-pandemic period since the availability of antigenic and molecular test results will probably be reduced.

In conclusion, wastewater surveillance of SARS-CoV-2 has proven to be a powerful tool for assessing disease incidence at the community level. However, it still needs to be integrated with other public health initiatives (e.g., campaign-based and randomized testing of individuals for the presence of pathogens or antibodies, clinical case reporting, and mobile-based contact-tracing and self-reporting systems) [50]. The greater reliability of wastewater SARS-CoV-2 RNA concentrations over clinically reported case counts is likely due to systematic biases that affect reported case numbers, including variations in access to testing and under-reporting of asymptomatic patients. With these advantages, namely, scalability and low cost, wastewater-based epidemiology can be a key component of public health surveillance for COVID-19 and other communicable infections. This represents a significant challenge considering the poor integration of the environmental and clinical science communities [51].

## Figures and Tables

**Table 1:** Characteristics of the wastewater treatment plants in place.

Wastewater Treatment Plant	Average Inflow (m^3^/die ± SD)	Served Population (N)
Agrigento	7593.0 ± 1238.6	55,000
Bagheria	9988.7 ± 1795.7	75,000
Caltanissetta	13,217.5 ± 2329.4	76,700
Enna	3807.7 ± 1170.2	34,000
Gela	1465.6 ± 475.9	12,000
Marsala	7500 ± n.d.	40,000
Mazara del Vallo	3630 ± n.d.	17,000
Messina	2488.9 ± 450.0	227,000
Modica	8061.8 ± 1940.3	50,000
Palermo WWTP1	83,205.2 ± 2292.1	53,886
Palermo WWTP2	19,438.7 ± 1575.0	314,973
Ragusa	11,171.6 ± 1725.7	58,000
Trapani	16,893.4 ± 2372.8	118,500
Vittoria	11,197.1 ± 2218.0	55,000
Total		1,187,059

**Table 2:** Correlation analysis between viral load in wastewaters, active cases, and severe clinical outcome.

		t0 Prevalence			t7 Prevalence			t14 Prevalence			Severe Clinical Outcomes		
	Time Periods (Months)	R	r^2^	*p*-Value	R	r^2^	*p*-Value	R	r^2^	*p*-Value	R	r^2^	*p*-Value
GC/L *	0–6	0.90	0.82	<0.001	0.89	0.79	<0.001	0.87	0.76	<0.001	0.93	0.61	<0.001
	7–12	0.77	0.59	<0.001	0.79	0.62	<0.001	0.69	0.47	<0.001	0.75	0.53	<0.001
	0–12	0.85	0.72	<0.001	0.87	0.76	<0.001	0.86	0.74	<0.001	0.90	0.51	<0.001

* GC/L: genomic copies/litre; R: correlation coefficients; r^2^: coefficient of determination; t prevalence: prevalence of active cases 0, 7, and 14 days after the sampling date of wastewater.

## Data Availability

All relevant data are included in the paper.

## Appendix A

SARI Collaboration group: Claudio Costantino (Department of Health Promotion, Mother and Child Care, Internal Medicine and Medical Specialties “G. D’Alessandro”, University of Palermo, Via del Vespro 133, 90127, Palermo, Italy); Arianna Russo, Gina Andolina, Viviana Giangreco, Francesca Rita Iaia, Arianna Santino, Rita Li Muli, Chiara Filizzolo, Mariangela Pizzo, Giuseppa Luisa Sanfilippo (Clinical Epidemiology Unit, Regional Reference Laboratory of Western Sicily for the Emergence of COVID-19, University Hospital “P. Giaccone”, Via del Vespro 133, 90127, Palermo, Italy), Annalisa Guercio, Francesca Gucciardi (Istituto Zooprofilattico Sperimentale della Sicilia “A. Mirri”, Via Marinuzzi, 90129, Palermo, Italy Istituto Zooprofilattico Sperimentale della Sicilia “A. Mirri”, Via Marinuzzi, 90129, Palermo, Italy), Alessandro Arrigo (Regional Health Authority of Sicily, Via Vaccaro 5, 90145, Palermo, Italy); Antonio Conti, Giuseppe Cuffari, Fabrizio Merlo (Regional Environmental Protection Agency (ARPA Sicilia), UOC “Reporting Ambientale, Salute e Ambiente”, Complesso Roosevelt, Viale Cristoforo Colombo snc, 90100, Palermo, Italy); Massimo Giuseppe Chiarelli, Andrea Polizzi, Giovanni Casamassima (Local Water Plant Management, Acque di Caltanissetta S.p.A., Corso Vittorio Emanuele 61, 93100, Caltanissetta, Italy); Mansueta Ferrara, Giuseppina Gullo (Local Wastewater Plant Management, Amap SpA, Via Volturno 2, 90100, Palermo, Italy); Roberto Ferlisi, Sabrina Merandi (Local Health Authorities, Via Enzo ed Elvira Sellerio, 45, 90100 Palermo, Italy).
